# No cancer left behind: a testbed and demonstration of concept for photoacoustic tumor bed inspection

**DOI:** 10.1080/24699322.2025.2604123

**Published:** 2025-12-31

**Authors:** Laura Connolly, Hyunwoo Song, Keshuai Xu, Anton Deguet, Simon Leonard, Gabor Fichtinger, Parvin Mousavi, Russell H. Taylor, Emad Boctor

**Affiliations:** a Department of Electrical and Computer Engineering, Queen’s University, Kingston, Canada; b Laboratory of Computational Sensing and Robotics, Johns Hopkins University, Baltimore, USA; c Department of Computer Science, Johns Hopkins University, Baltimore, USA; d School of Computing, Queen’s University, Kingston, Canada

**Keywords:** Photoacoustics, robotics, breast-conserving surgery, margin detection

## Abstract

Cancer resection surgery is unsuccessful if tumor tissue is left behind in the surgical cavity. Identifying the residual cancer requires additional imaging or postoperative histological analysis. Photoacoustic imaging can be used to image both the surface and depths of the resection cavity; however, its performance hinges on consistent probe placement and stable acoustic and optical coupling. As intra-cavity deployment of photoacoustic imaging is largely uncharted, several potential embodiments warrant rigorous investigation. We address this need with an open-source robotic testbed for intraoperative tumor-bed inspection using photoacoustic imaging. The platform integrates the da Vinci Research Kit, depth imaging, and electromagnetic tracking to automate cavity scanning and maintain repeatable probe trajectories. Using tissue-mimicking phantoms, we (i) demonstrate a novel imaging embodiment for photoacoustic tumor-bed inspection and (ii) show how this testbed can be used to investigate and optimize tumor bed inspection strategies and configurations. This study establishes the feasibility of detecting and mapping residual cancer within a simulated surgical cavity. The primary contribution is the testbed itself, designed for integration with existing surgical navigation workflows and rapid prototyping. This testbed serves as an essential foundation for systematic evaluation of photoacoustic, robot-assisted strategies for improving intraoperative margin assessment.

## 1. Introduction

Achieving complete tumor resection during cancer surgery is often challenging because tumor tissue may be non-palpable, irregularly shaped or have nonlinear borders. Breast-conserving surgery is a surgical procedure where this challenge is commonly encountered. During these procedures, the surgeon resects the tumor tissue as well as some of the surrounding breast tissue to prevent disease recurrence while maintaining breast cosmesis. Surgical margins, indicating the presence or absence of residual cancer tissue within the surgical cavity, are analyzed post-operatively by a pathologist. Approximately 30% of these procedures result in positive margins, which indicate that residual cancer is left behind on the tumor bed, and require immediate repeat surgery [1].

In many cases, as a general precaution to prevent positive margins and reduce the risk of re-operation, the surgeon will perform an indiscriminate and coarse shave of the entire cavity after tumor resection [2]. In the context of breast conservation especially, this additional shave can contribute to post-surgical cosmetic deformation. Providing technical means for identifying residual cancer in the cavity to alert the surgeon when this additional shave is necessary could therefore help preserve healthy tissue in patients where a shave is not necessary.

There are several intraoperative or perioperative imaging techniques that are currently under investigation for margin assessment [3]. Some of these techniques include mass spectrometry imaging during resection [4], raman spectroscopy scanning of the resected specimen [5], or fluorescence imaging of the tumor bed [6]. Each of these approaches suffer from various shortcomings with respect to feasibility. For instance, the ‘iKnife’, a mass spectrometry device, relies on additional tissue incineration to identify positive margins and can therefore potentially contribute to unnecessary healthy tissue loss. Similarly, the ‘MarginBot’ is used to analyze excised tissue, assuming the tumor bed is aligned with the resected specimen borders [5]. This assumption is not always true and often tissue that is presumed to be left behind, was actually incinerated at the specimen border. Fluorescence imaging is also limited by the need for a direct line of sight to the target anatomy and may have constraints in detecting subsurface residual cancer. Considering these limitations, an ideal margin detection solution should be noninvasive, able to image both surface and subsurface tissue, and be deployable within the surgical cavity to reflect the true status of the tumor bed.

Photoacoustic imaging is an emerging biomedical modality that, unlike pure optical imaging techniques, can maintain resolution in deep tissue [7]. This approach relies on pulsed laser excitation of tissue that induces thermoelastic expansion and creates an acoustic wave [8]. This phenomena is referred to as the photoacoustic effect. Just as with ultrasound imaging, the acoustic data is then processed using beamforming techniques to create a photoacoustic image [9]. Furthermore, biomarkers or constrast agents can be used to target specific anatomy, in-vivo [10]. These agents are used to alter the photoacoustic characteristics of local tissue and are typically in the form of small-molecule dies or nanoparticles such as carbon and gold [11]. These contrast agents are being actively investigated for targeted cancer imaging. For example, Wu et al. showed that prostate-specific membrane antigen can be targeted with contrast agents and imaged with photoacoustics to isolate prostate cancer [12]. It has been demonstrated that these same target molecules are also expressive in neoplastic breast tissue [13]. Multiple studies suggest that these contrast agents will become more widely available and adaptable to different biological targets as the clinical use of photoacoustic imaging becomes more prevalent and further in-vivo validation is conducted [11,14,15]. Although photoacoustic imaging of breast cancer has been explored extensively [16,17], most of the literature is focused on whole breast imaging [18,19]. The studies that have used photoacoustics for margin detection use scanners to image the *ex vivo* specimen [20,21] or use an *in vivo* probe where the light source and acoustic detector are co-located and the user has to maintain contact and pressure with the tissue for acoustic coupling [22–24]. To our knowledge, there has been no published research on photoacoustic imaging with a decoupled light source and detector for intra-cavity margin detection in breast-conserving surgery.

Deploying an imaging device to localize and identify residual cancer on the surface of the tumor bed is made difficult by the nature of the surgical cavity in breast-conserving surgery, which is often confined, deformable and dynamic. Photoacoustics allows flexible, fiber-delivered excitation and only requires an acoustic coupling path for detection, however, image quality is highly sensitive to the relative source-detector geometry. Because multiple embodiments are possible for photoacoustic tumor bed inspection, systematic experimentation is needed to determine the optimal source–detector pose for a given cavity and target. These sensitivities make automation valuable during experimentation, to enable repeatable trajectories and systematic parameter sweeps. Automation will also be essential to ensure sufficient scanning coverage and precise localization to indicate when and where a cavity shave is necessary. Furthermore, there has been a broader shift toward robot-assisted operative workflows, making automation not only useful for experimental repeatability but also directly relevant to clinical translation. For example several centers have used the da Vinci robot (Intuitive, USA) to provide improved visual access to the vasculature to prevent nipple necrosis during mastectomy [25,26].

As the use of photoacoustics for intra-cavity imaging is unexplored, there are no available testbeds or development platforms to build on. To properly exhaust the potential configurations, a testbed that facilitates automation, navigation, and 3D visualization is necessary. Therefore, in this paper, we introduce an open-source testbed and demonstrate a potential embodiment of photoacoustic inspection of the tumor bed for breast-conserving surgery. Unlike previous works [27–29], where the light source and detector are co-located in a single probe, we decouple these to enable intra-cavity inspection that does not require direct contact with the tumor bed. We assess our proposed strategy on simulated tumor bed models and show that we can detect simulated residual cancer while uncovering the limitations and future directions for research. To our knowledge, this is the first demonstration of photoacoustics for margin assessment in this capacity [30] and the only experimental testbed available for future research. Although we present this method in the context of breast surgery, in theory, it can be applied to other cancer resection procedures such as partial nephrectomy [31] or partial hepatectomy [32]. This paper is positioned to serve as an essential foundation for subsequent, more extensive investigations for photoacoustic tumor bed inspection.

## 2. Materials & methods

### 2.1. Overview

**Figure 1.**
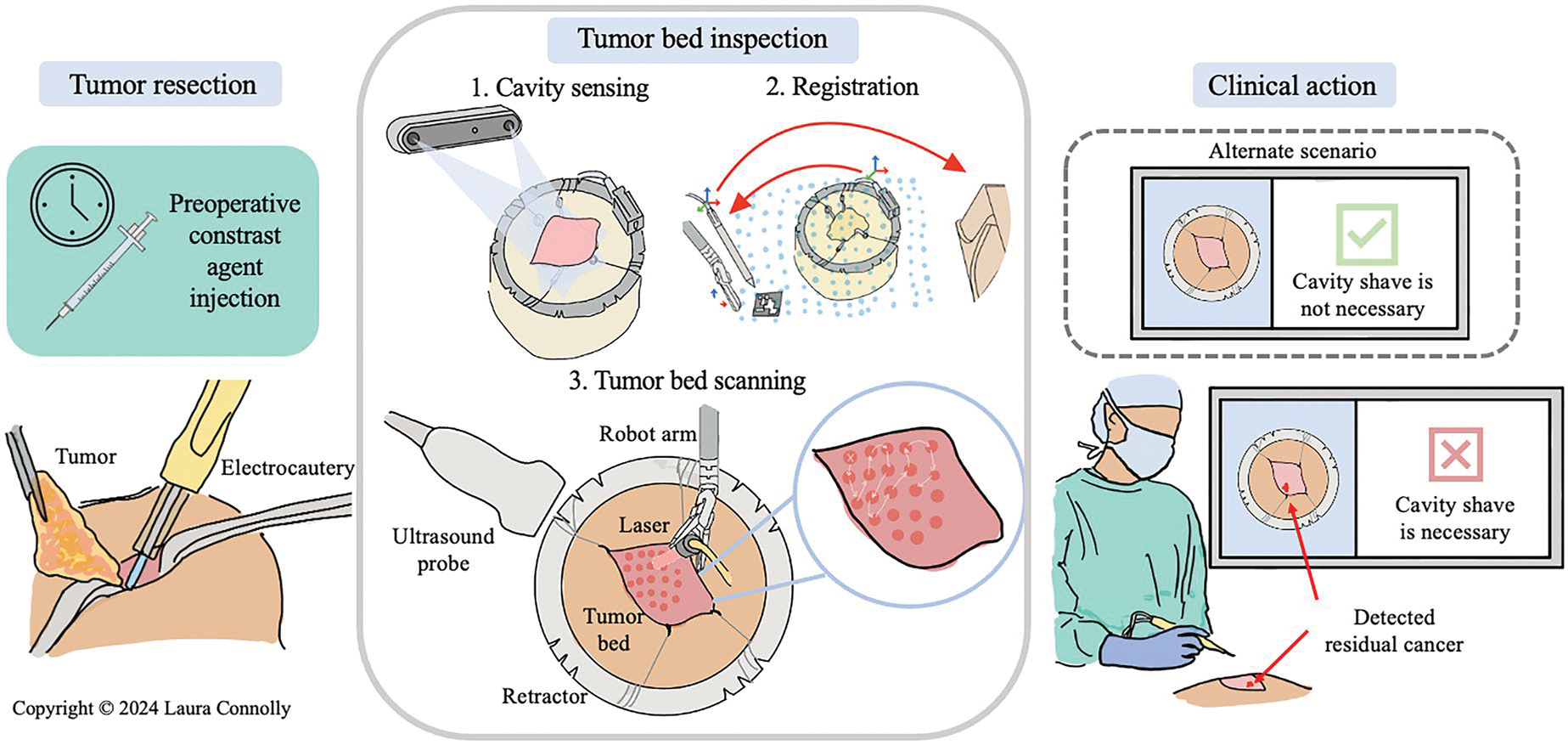
Overview of proposed approach demonstrating pipeline of contrast agent injection to clinical action.The workflow steps for the testbed (cavity sensing, registration and scanning) are highlighted in the center of the figure. In future iterations of this work, the efficiency of these steps would be automated to ensure that we do not significantly disrupt the surgical workflow. (Figure copyright © Laura Connolly, used by permission).

The overall workflow for our proposed method is shown in Figure 1 below. First, a targeted photoacoustic contrast agent is injected systemically into the patient before surgery. Once the agent has been uptaken by the targeted tumor tissue, tumor resection proceeds as usual. After the tumor is resected, a retractor is used to prop open the surgical cavity to reveal the tumor bed. Subsequently, tumor bed inspection is deployed using the da Vinci surgical robot. Our workflow for deploying tumor bed inspection can be categorized into three phases: cavity sensing, registration, and tumor bed scanning. These phases are described in more detail in the following sections. If residual cancer is detected, the surgeon can then perform a cavity shave to remove any residual tumor tissue that was initially missed.

### 2.2. Tumor bed model

**Figure 2.**
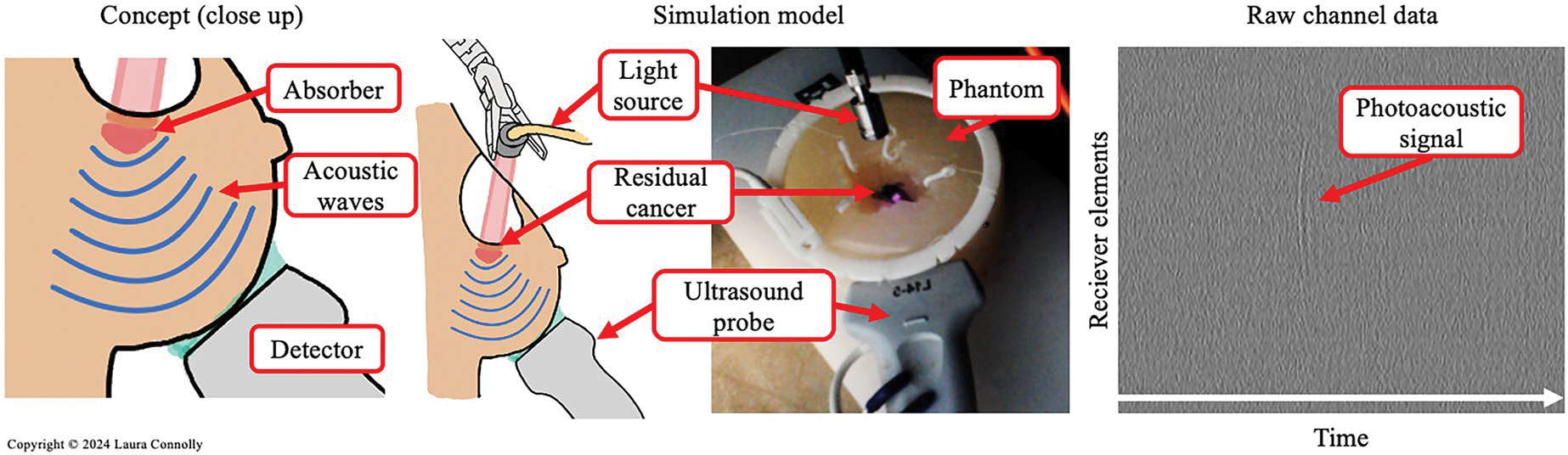
*Left:* Concept for photoacoustic inspection highlighting the decoupled light source and acoustic detector. *Middle:* Simulation model (diagram and captured image). *Right:* Raw channel data from ultrasound probe demonstrating the binary signal indicator. (Figure copyright © Laura Connolly, used by permission).

To demonstrate this imaging approach and testbed, we simulated the conditions after breast-conserving surgery. The photoacoustic effect is induced when an absorber is illuminated and undergoes thermoelastic expansion. During this expansion, sound waves are emitted which can be detected by an acoustic receiver. In our proposed approach, the absorber is the residual cancer that has uptaken the photoacoustic constrast agent. In our simulation model, we mimic this absorption behavior with India Ink that is inserted into a breast phantom (Figure 2). Upon laser excitation, the ink induces the photoacoustic effect which can be detected by an ultrasound probe that is fixed to the outside of the phantom. The raw channel data, shown in Figure 2 on the right, can then be used as a binary indicator as to whether or not the material under inspection has induced the photoacoustic effect.

The healthy tissue in our simulation model, is made up of a combination of plastisol, which is liquid plastic that can be cast under heat, and cellulose, which is a powder that can be added to ensure ultrasound scattering. This material does not induce a photoacoustic effect. To simulate a tumor bed, the site where a tumor was previously located, we carved a cavity into three plastisol phantoms after they had hardened. The resection cavity in each phantom is approximately 4–5 cm wide with simulated residual lesions around 3 cm in size. These parameters were selected to reflect realistic clinical conditions where patients who undergo breast-conserving surgery generally have tumors that are ≤ 3 cm [33,34]. The resection cavities are designed to represent a resection of tumors in such size with a conservative margin but also to provide ample tissue surface for validating the testbed and proposed imaging technique. The ‘residual tumor tissue’ / India ink absorber was then mixed with Barium Sulfate and painted onto the surface of the tumor bed. Barium Sulfate was added to the residual cancer mixture because it does not induce a photoacoustic effect but is visible under Computed Tomography (CT) imaging. This mixture was sealed into the phantoms by adding a thin layer of plastisol on top after it had dried. To obtain a ground truth, each of the phantoms was imaged using the Loop-X system by Brainlab (Munich, Germany) which is a cone beam CT machine. Intraoperative CT is not typically available during breast-conserving surgery procedures, therefore the CT images and volume reconstructions were just used for experimental evaluation.

### 2.3. Testbed

**Figure 3.**
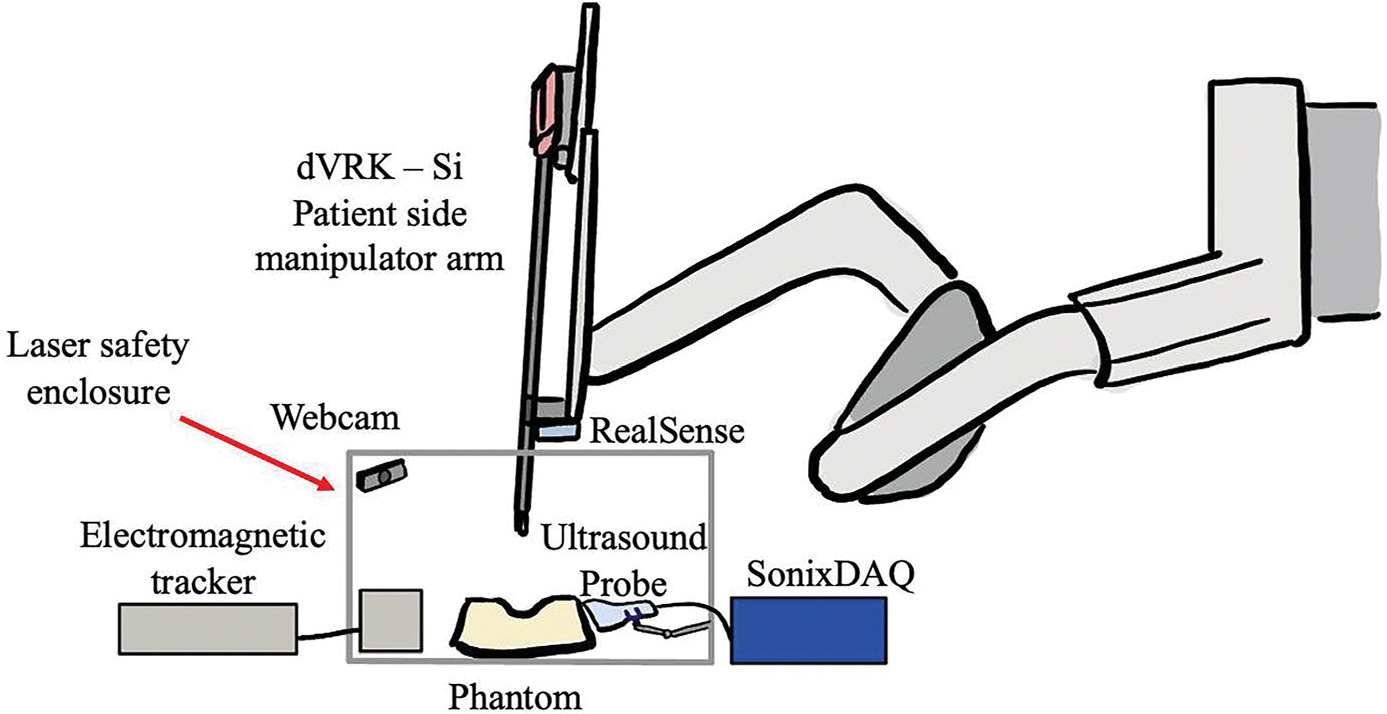
Labeled overview of testbed showing the laser safety enclosure.

To construct a testbed for this system, we rely on a combination of accessible technology and open-source software. The full list of equipment for this study includes: a da Vinci Research Kit (dVRK) Patient Side Manipulator (PSM) [35], an Intel RealSense RGB-D Camera (Intel, USA), an Ascension 3DG EM Tracker (NDI, Canada), a 3D-printed retractor fit with an EM sensor, an 808 nm laser (AeroDiode 808LD-4–0-0, France), an Ultrasonix L14–5 ultrasound probe (BK Medical, USA), a SonixDAQ (BK Medical, USA), a Windows laptop, and a Linux desktop. The experiment was also conducted under a laser safety enclosure because the wavelength of the laser is invisible to the naked eye and can pose a serious hazard to the operator (Figure 3). To effectively monitor the scanning and ensure it was safe to make modification inside of the enclosure (i.e. the laser was off and pointing in a safe direction), we also mounted a webcam inside of the enclosure facing the phantom. Data processing and acquisition for this project is all done using the open-source medical imaging platform, 3D Slicer [36]. Data from the EM tracker and Intel RealSense camera is streamed into 3D Slicer *via* PLUS Toolkit [37]. Communication with the dVRK-Si is facilitated with SlicerROS2 [38] and the ROS2 wrapper for the dVRK (https://github.com/jhu-dvrk). Raw ultrasound data is streamed from the SonixDAQ to 3D Slicer using OpenIGTLink [39] and the pyigtl python library. To account for operating system incompatibilities between device drivers, a peer-to-peer network is established between the Windows laptop, which hosts PLUS and OpenIGTLink connections, and the Linux PC, which hosts the dVRK-Si connection.

#### 2.3.1. Cavity sensing

**Figure 4.**
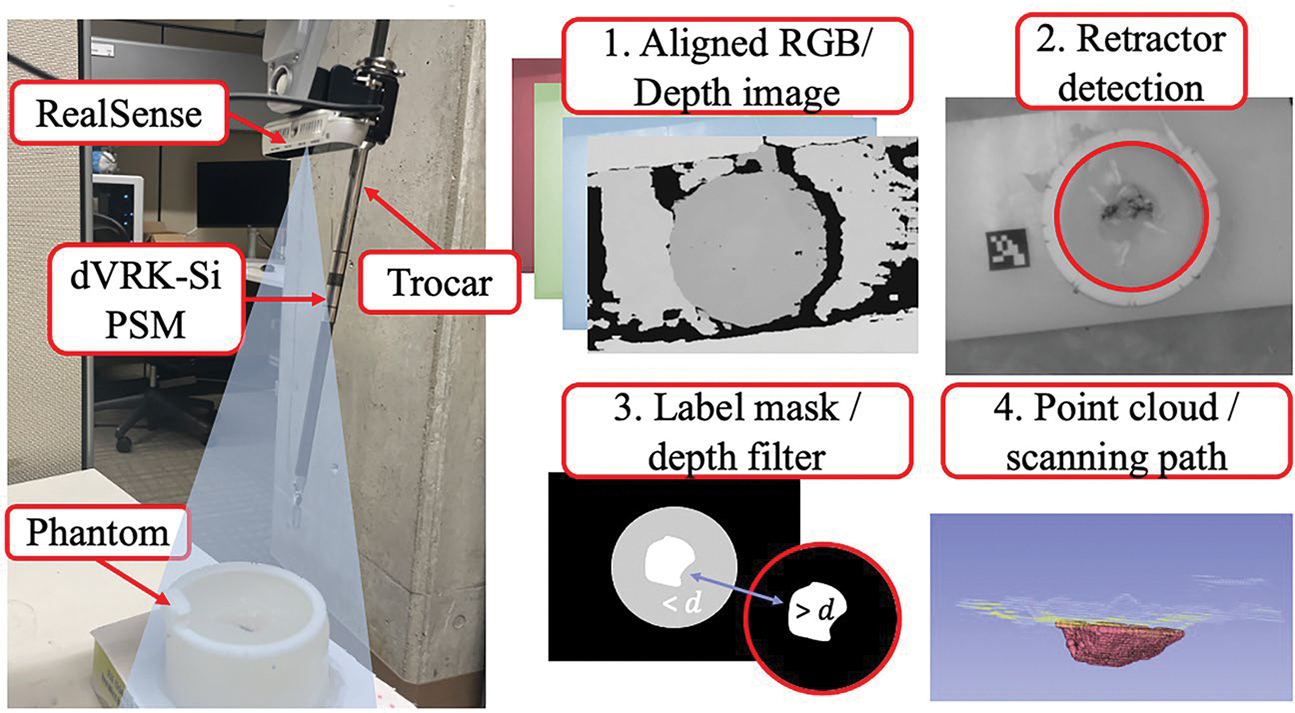
*Left:* RealSense and dVRK configuration. *Right:* Filtering steps for sensing the surgical cavity. Depth threshold is denoted at *d* to illustrate the depth filtering in step 3. (Figure copyright © Laura Connolly, used by permission).

To detect the surgical cavity, the RealSense camera (which is fixed to the dVRK PSM) is used to capture an image from above the phantom. The data from the RealSense, which is the aligned RGB and depth image, is streamed into 3D Slicer and converted into a point cloud using the DepthImageToPointCloud module by Barr et al. [40] (https://github.com/PerkLab/DepthImageToPointCloud). To segment the tumor bed from the surrounding tissue and area, we rely on a series of image filters (Figure 4).

We apply a canny edge detector and hough transform to identify the retractor in the image. The inside of this circle is then used as a label mask for the aligned depth image. From there we use a simple depth threshold to segment the tumor bed from the surrounding tissue within the retractor. After thresholding, we isolate the points in the depth cloud that correspond to the tumor bed and stream these separately into 3D Slicer using the aforementioned module. The points from this depth cloud are used as our scanning path. It should be noted that there are more sophisticated approaches for obtaining a 3D point cloud of the anatomy that will be integrated into future work for reliable clinical translation [41,42].

A resolution of 3 pixels per point was set as the density of the tumor bed point cloud. The point cloud is also manually registered to the CT of the phantom for validation. This registration is performed using specific landmarks from each cavity that are visible in a volume reconstruction of the CT and the point cloud (for example the phantom shown in Figure 4 would be aligned based on the sharp ridge shown on the bottom of the cavity in step 3). Each phantom had a distinct and nonsymmetrical shape with various landmarks that were prevalent in the point cloud and volume reconstruction of the CT which made manual registration possible. In future studies, CT beads and registration landmarks should be used to automate this part of the workflow.

#### 2.3.2. Registration

**Figure 5.**
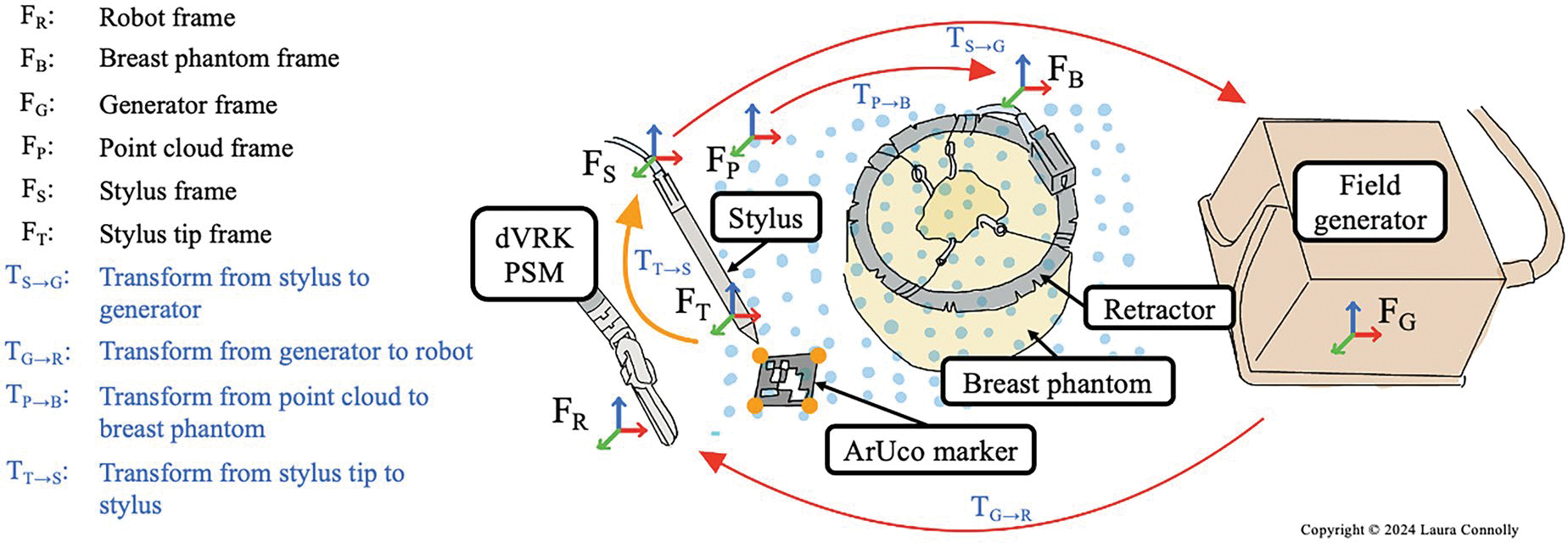
Coordinate systems and transformations used during tumor bed inspection. (Figure copyright © Laura Connolly, used by permission).

There are several coordinate frames involved in this experiment (Figure 5). The frames of particular interest are: the point cloud coordinate frame ($F_{P}$), the EM coordinate frame which is measured with respect to the position of the field generator ($F_{G}$), and the robot coordinate frame which lies at the remote center of motion (RCM) of the PSM ($F_{R}$). We also assume that an EM sensor that is embedded into the retractor can be considered the coordinate frame of the breast phantom ($F_{B}$) and account for the relative motion of the phantom during scanning. A series of point-to-point registrations are performed to align these coordinate frames using the ‘Fiducial Registration Wizard’ module in SlicerIGT [43,44]. Fiducial Registration Wizard uses the Horn Method to compute the necessary transform to match two sets of landmarks [45].

For each of the following registrations, we use the four corners of an ArUco marker that is fixed to the table beside the phantom as our registration points. The points are first identified in the RGB image and then transformed into the point cloud coordinate system ($F_{p}$) using the following equations:

$$z=\text{depthImage}\left(i,j\right)$$

$$x=\left(i-P_{p}\left(x\right)\right)\cdot \frac{z}{f_{i}}$$

$$y=\left(j-P_{p}(y)\right)\cdot \frac{z}{f_{i}}$$

Such that i and j are the pixel coordinates of the depth image, $P_{p}(x)$ is the principal point of the camera in the x direction, $P_{p}(y)$ is the principal point of the camera in the y direction, and $f_{l}$ is the focal length of the camera. This methodology follows that of the DepthImageToPointCloud module mentioned earlier.

To transform the point cloud into the EM coordinate system, we first start with a pivot calibration of the EM-tracked stylus to obtain ($T_{T\to S}$). This is done using the ‘Pivot Calibration’ module in the SlicerIGT extension [44]. The corners of the ArUco marker are then captured with the tracked stylus relative to the $F_{B}$ frame. The ‘Fiducial Registration Wizard’ in SlicerIGT is then used to obtain the transformation $T_{P\to B}$ using a point to point registration based on the position of the ArUco corners in $F_{P}$ and $F_{B}$ This module provides a simple interface for performing landmark registration based on information in the 3D Slicer scene. We also note that the position of the retractor sensor with respect to the generator is streamed directly into 3D Slicer with the PLUS toolkit. After this registration, we have successfully aligned the point cloud coordinates into the coordinate system of the EM sensor that is embedded in the retractor (step 1). The average root mean squared error for this step was 2.8 mm.

For the final registration, the same stylus is used to touch the corners of the ArUco marker while it is tracked with respect to the generator $F_{G}$. The corners of the ArUco marker are also captured in the $F_{R}$ coordinate system by manually guiding the dVRK to each corner. To capture these coordinates, the measured cartesian position of the device, provided by the ROS2 interface for the dVRK, is streamed into 3D Slicer using SlicerROS2. At this stage the control point of the dVRK PSM is also modified such that it represents the tip of the end-effector jaws (rather than the default position at the center of the jaws). Again, ‘Fiducial registration Wizard’ is used to obtain $T_{G\to R}$ based on these point locations. After these transformations are generated, the ‘Data’ module in 3D Slicer is used to setup the correct hierarchy such that the point cloud observes $T_{P\to B}$, and the EM frames observes $T_{G\to R}$. After this, we have successfully aligned the EM coordinate system to the robot coordinate system (step 2). The average root mean squared error for this step was 1.2 mm.

#### 2.3.3. Tumor bed scanning

**Figure 6.**
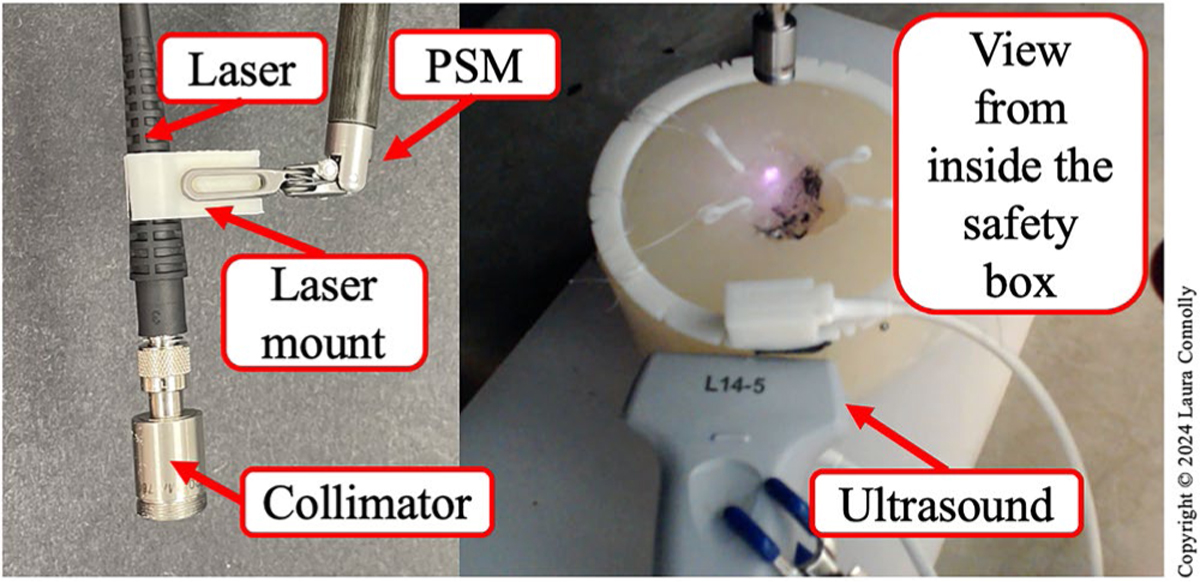
*Left:* Labeled light source with mount and collimator. *Right:* View inside the safety enclosure. (Figure copyright © Laura Connolly, used by permission).

After cavity sensing and registration, we initiate tumor bed scanning. To do so, the laser is fixed to the end-effector of the dVRK using a 3D-printed mount. A collimator is also attached to the end of the optical fiber to project a collimated 2 mm diameter beam with negligible beam width variation in normal operating condition (Figure 6 - left). The laser safety enclosure, which has an insertion point at the trocar of the dVRK (labeled in Figure 4), is placed around the field which now includes the EM field generator, tumor bed model, ultrasound probe, PSM with the laser mount and a webcam to monitor the scanning (Figure 6 - right). The webcam is necessary for monitoring the status of the scanning during validation to ensure that nothing unexpected occurred. We feel that this is essential for testbed development, particularly when the developer is modifying the scanning pattern or technique.

The control point of the dVRK is then modified to reflect the goal position of the laser spot while maintaining some height between the collimator and the surface of the bed. As each of the coordinate systems are registered to the robot’s coordinates, we monitor the live position of the scan point using the encoder data from the PSM that is streamed over SlicerROS2.

Finally, the ultrasound probe is fixed to the outside of the phantom using a workbench mount (Figure 6 - Right). The position of the ultrasound probe was selected based on the 3D navigation view and visual inspection. To obtain the position of the tracked image, we performed tracked pointer ultrasound calibration, demonstrated in [46]. This calibration was done using an EM sensor fixed to the end of the probe and by swapping the SonixDAQ for the standard cart to obtain the B-mode ultrasound images over PLUS and OpenIGTLink. As with the other registrations, this calibration was performed using SlicerIGT. Based on the position of the tracked image, we selected an ultrasound orientation that appeared to intersect with as many scan points as possible. We recognize that parameter of the experiment is highly dependent on the operator and should be optimized in future iterations of this research. The ultrasound probe has a frequency range of 5–14 megahertz, a focal range of 20–90 mm and 128 elements. After the probe is fixed, a python script is used to command the dVRK to each point on the surface of the tumor bed in 3 s increments, during which the raw ultrasound data from the DAQ is also captured. The data is recorded using the ‘Sequences’ module in 3D Slicer and analyzed at each time point to determine whether or not a photoacoustic signal is present. When a signal is identified, the position of the laser point is noted as a positively detected residual cancer point and marked with a red fiducial the 3D viewer.

As it is unlikely that a surgeon will resect residual cancer at a pixel-wise resolution, we also came up with a different evaluation metric we call quadrant localization accuracy. This metric is a reflection of how well we can localize residual cancer to a particular region of the cavity and is computed by dividing the scanned point cloud into four equal quadrants. If residual cancer is detected in a quadrant where there are pixels in the aligned CT that contain contrast, we consider this localization successful.

## 3. Results & discussion

**Figure 7.**
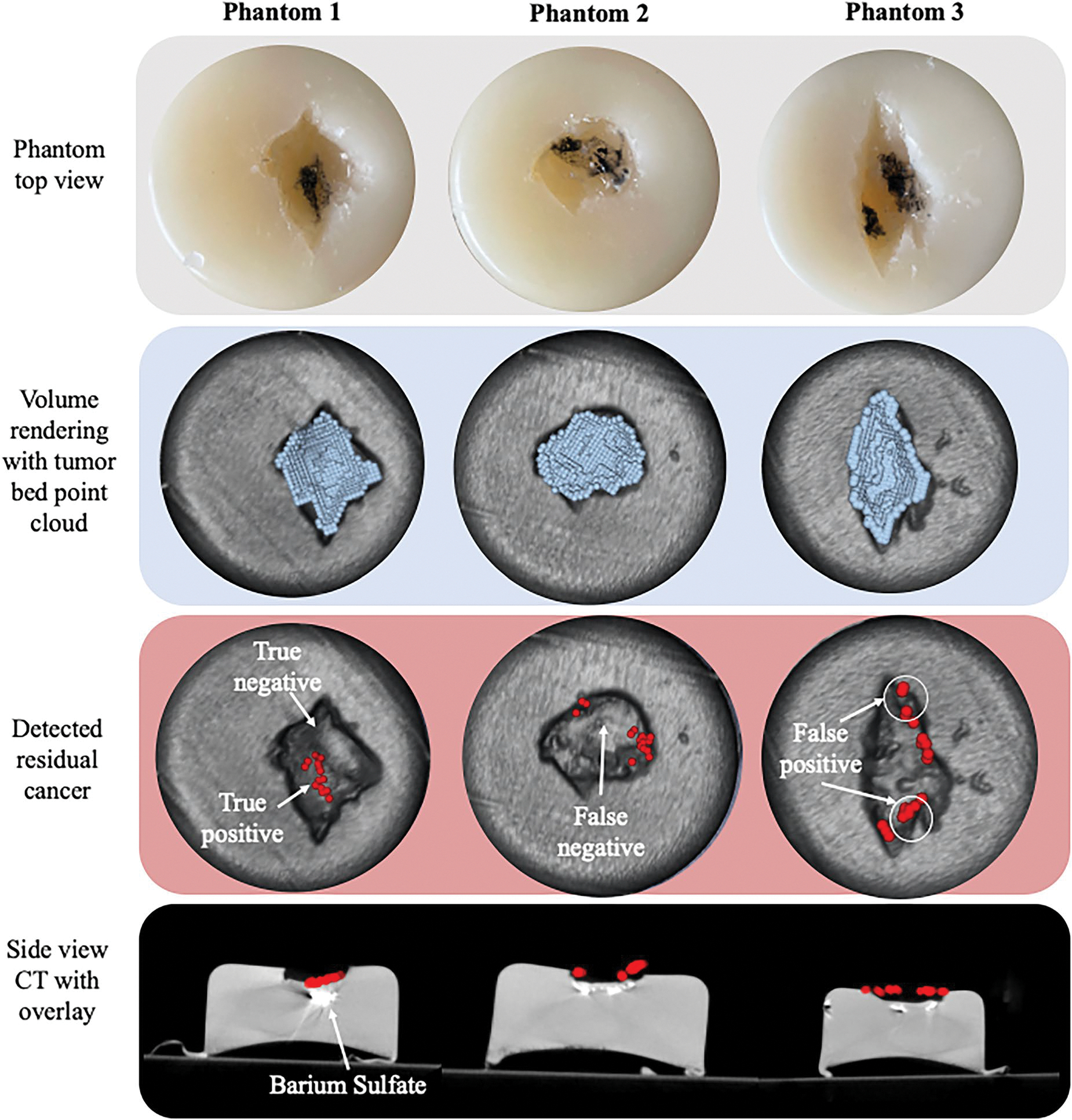
*First row* – top view of phantoms. *Second row* – tumor bed point cloud overlaid on volume reconstruction of CT. *Third row* – Positively identified residual cancer overlaid on volume reconstruction. *Fourth row* – Cross sectional CT views with projection of detected residual cancer. Quantitative evaluation metrics are displayed below each phantom. (Figure copyright © Laura Connolly, used by permission).

Figure 7 below demonstrates the results of tumor bed inspection on each phantom with the ultrasound probe in a single fixed location. The point cloud of each segmented cavity is also overlaid on a volume reconstruction of the phantom CT to show the output of the cavity sensing step.

As shown in Figure 6, row 2, the proposed cavity sensing method can successfully segment a sufficient region of the tumor bed in each phantom. This can be confirmed by visual inspection of the overlap between the detected cavities and the volume reconstruction from each phantom. Small inconsistencies are visible on the boundaries of the point clouds, where the detected point cloud does not overlap entirely with the cavity in the volume reconstruction. These inconsistencies may lead to potentially missed residual cancer at the periphery of the tumor bed. However, the tissue on the borders is adjacent to the incision site and typically distanced from the breast lesion. Therefore, the likelihood of detecting residual cancer in this area is low. These inconsistencies can also likely be resolved with a higher resolution camera or a higher point cloud density. It is important to note that the point cloud density (3 pixels per point) was selected to accommodate both scanning time and sufficient coverage because of the slow acquisition speed of the SonixDAQ and need to stop for 3 s at each point to ensure the PA signals are separable. In total, the length of time required for system setup, cavity, sensing, and scanning, is also highly dependent on the operator. For these experiments, it took approximately 30 min to prepare for each scan, and an average of 16.5 min per scan. These elements could be optimized with programming workflows and path scanning optimization.

**Figure 8.**
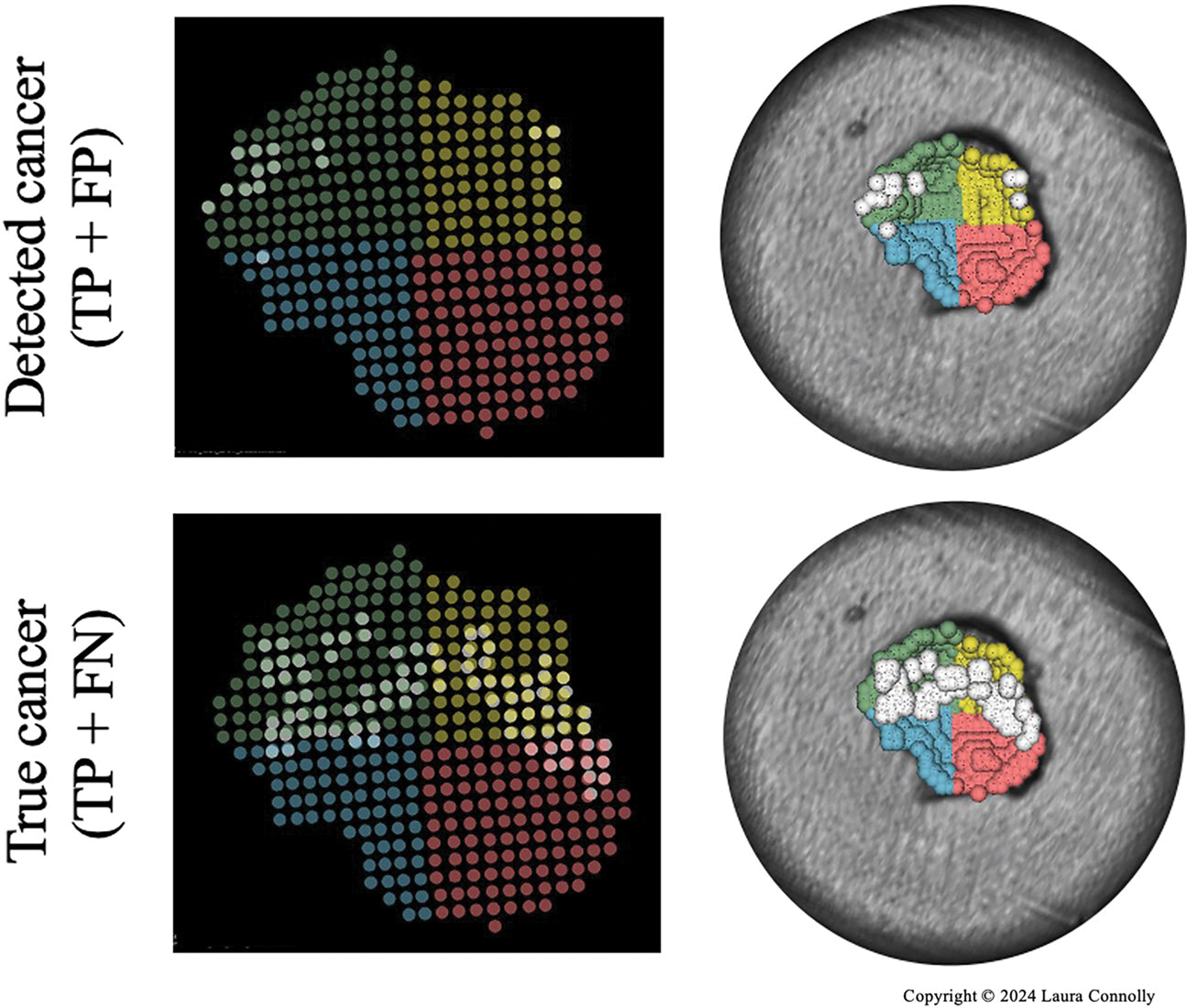
Visualization of quadrant localization for Phantom 2. Each quadrant is shown in a different color on both the 2D projection of the cavity point cloud and the overlay on the volume rendering. The top row images demonstrate the location of detected residual cancer (i.e. true and false positives) in white. The bottom row images demonstrate the true location of residual cancer in white (i.e. true positives and false negatives). (Figure copyright © Laura Connolly, used by permission).

In row three, the positively identified residual cancer regions are shown in red overlaid on the volume reconstruction of the CT. These can be compared to the top view of each phantom in row 1, where black ink represents the ground truth residual cancer locations. In each case, photoacoustic imaging detected some residual cancer in the appropriate regions. This is particularly evident in Phantom 1, where the residual cancer is concentrated in the center of the tumor bed. The inspection method could also identify residual cancer on Phantom 2, where it is detected on both the edges of the tumor bed. In Phantom 3, there are some false positives (indicated in Figure 7) which are likely the result of inaccurate laser / robot positioning or tracking inaccuracy. A close up of the quadrant localization accuracy for one of the phantoms (Phantom 2) is also shown below in Figure 8.

Using the same technique for each phantom, we were able to successfully localize residual cancer in at least 3 out of 4 quadrants (with a quadrant localization accuracy of 75%, 75% and 100% for phantoms 1, 2 and 3 respectfully). In each of these phantoms, the outcome of the scan would be that residual cancer is detected and cavity shave is necessary. Even in scenarios where the localization is not perfect, like phantom 3, the desired outcome is achieved.

For each phantom, we also include the 2D projection on the cross-sectional view of the CT in row 4. Please note that the detected residual cancer points are projected onto a particular cross-section of the CT for simplification. In areas where the points appear to be above the surface, it is the result of height differences along the tumor bed. In each phantom, there are positively identified residual cancer points above subsurface Barium Sulfate, our ground truth. This suggests that our inspection method can positively identify underlying residual cancer beneath the surface of the tumor bed. Therefore, the penetration of the laser is sufficient for inducing the photoacoustic effect on subsurface residual cancer. Although we do not apply beamforming to do true photoacoustic imaging in this work, extending this method to further identify the extent of residual cancer (in depth) would simply require tracking the ultrasound probe and applying segmentation. We also observe several false negatives in these scans i.e. areas where there is residual cancer visible on the phantom but no photoacoustic signal (indicated in Figure 7). These are likely the result of the ultrasound probe positioning. The elevational focusing of the ultrasound probe and the direction in which the acoustic wave propagates through the medium are important factors as to whether or not a photoacoustic signal will be detected. Previous studies have shown that a change in the direction of the ultrasound probe of as little as 5 degrees, can result in a drop in signal intensity of more than 50% [47]. This also accounts for the false negatives observed for each of these phantoms because the utlrasound probe is fixed in a singular location during scanning that may not have provided the necessary field of view to capture the photoacoustic signal at each scan point. This is an inherent limitation of the non-contact configuration that is not encountered on systems where the probe and light source are coupled and in the same imaging plane. In other embodiments of this concept, a wobbler or 3D ultrasound can be used or the ultrasound probe can robotized to keep the laser spot within the ultrasound plane [48]. For this embodiment however, this limitation does not impact the capacity of the system to detect residual cancer and indicate when a cavity shave is necessary.

We note that one of this work is that experimentation is conducted on phantoms rather than human tissue. There are no accurate animal models for simulating human breast tissue, and cadaveric samples are unusable due to their rapid rate of tissue necrosis. The phantoms designed for this experiment were made using the standard materials for ultrasound-compatible breast models, however, these models do not encapsulate all of the material properties of breast tissue [49]. In particular, one of the limitations of these models is that the simulated tissue is more rigid than real breast tissue. However, we assume that in a real clinical scenario, the retractor that is used to prop open the cavity will constrain the motion of the tumor bed. Moreover, because the laser does not come in contact with the tissue under inspection, we do not expect any additional surface deformation. If the contact pressure of the extracorporeal ultrasound probe causes any shifting, the EM sensor embedded in the retractor can capture the relative motion of the cavity. Motion and potential deformation can also be caused by the respiratory motion of the patient’s chest. One of the benefits of the embodiment described in this paper is that the robot used to guide the laser can also be used to enforce motion compensation. Another limitation of the phantoms is that they are more homogeneous than natural breast lesions and surrounding tissue. In future translation, multispectral photoacoustic decomposition should be applied, where multiple wavelengths of light are used in addition to the photoacoustic effect to isolate targeted tissue. This method has been investigated in detail in other studies [10]. Despite these practical limitations, these preliminary phantom results demonstrate the feasibility of our tumor bed inspection approach and the utility of the testbed. They serve as an essential foundation for future *in vivo* studies that are necessary for clinical translation.

Lastly, ongoing research at Queen’s University has demonstrated the potential of using EM tracking for navigation in breast-conserving surgery procedures during tumor resection [50]. Several of the tools and technology that are used for this testbed are already being used in these studies. Given these commonalities, after obtaining the positively detected residual cancer locations, the surgeon can simply use an EM-tracked cautery that is already integrated into navigated breast surgery to extract this tissue. Our margin detection method can therefore be smoothly integrated into these existing, and clinically validated, workflows.

## 4. Conclusions and future work

In this paper, we present a testbed for rapid prototyping and automation of photoacoustic tumor bed inspection. We use this testbed to demonstrate a potential system embodiment using a decoupled light source and extracorporeal acoustic detector. Phantom experiments established feasibility for detecting residual disease and surfaced limitations of the selected configuration, informing concrete directions for improvement. Although the results of this experiment are promising, more embodiments and configurations should be exhausted and explored using our testbed. Additionally, one of the limitations of this work is that the tumor bed phantoms are not representative of real human tissue; therefore, future work will be focused on animal studies to facilitate pre-clinical translation.

The ability to detect residual cancer intraoperatively has the potential to significantly reduce the presently high rate of positive margins in breast-conserving surgery procedures. Photoacoustic imaging is a strong candidate for this task because it provides both surface and depth tissue detection, with resolution set by the selected light source, thereby enabling mesoscopic detection of targeted tissue. By establishing an automated, cavity-focused photoacoustic workflow, this testbed lays the groundwork to reduce re-excisions and make margin assessment faster and more reliable.

## Data Availability

We are working on making the data used in this study available.
